# Feasibility of implementing Dignity Therapy in Dutch nursing homes: A pre–post study exploring potential effects on dignity, depression, and self-esteem

**DOI:** 10.1017/S1478951526101746

**Published:** 2026-02-04

**Authors:** Herman Van Dammen

**Affiliations:** Department of Anesthesiology, Pain and Palliative Medicine, Radboud University Medical Center, Nijmegen, The Netherlands

**Keywords:** Dignity therapy, nursing home residents, depression, self-esteem, feasibility

## Abstract

**Objectives:**

Dignity is a crucial value in caring for nursing home residents. These residents are extremely vulnerable due to, among others, their physical, social, and mental health risks. These risk factors can undermine their sense of dignity and induce feelings of inferiority and even depression.

Dignity Therapy is a short, individualized psychotherapy aimed at decreasing the existential distress of patients with a terminal illness. It appeared to be successful in patients with incurable cancer and could be a valuable addition to the treatment of loss of dignity in nursing homes. We evaluated the feasibility of implementing Dignity Therapy in Dutch nursing homes and explored its potential effects on residents’ dignity, depression, and self-esteem.

**Methods:**

A pre–post feasibility study was conducted in 2 nursing homes. Psychologists were trained to recruit residents and deliver Dignity Therapy. Standardized questionnaires were administered at baseline and follow-up to assess dignity, depressive symptoms, and self-esteem.

**Results:**

Psychologists were able to recruit and deliver the intervention to 36 residents. Participants generally evaluated the experience as pleasant and meaningful. No significant differences were found between pre- and post-measurements for dignity, depressive symptoms, and self-esteem. Regarding depressive symptoms, men and non-religious residents showed higher levels of depressive symptoms after the 8-week follow-up.

**Significance of results:**

Dignity Therapy is feasible and acceptable for residents in Dutch nursing homes. Although no significant effects on dignity, depression, or self-esteem were detected, further research with larger samples and optimized implementation strategies is needed to understand the potential impact of Dignity Therapy in this setting.

## Introduction

During the COVID-19 pandemic, it became clear that mental health issues are particularly evident in nursing homes (Cortés Zamora et al. 2022). Due to the physical decline of these vulnerable nursing home residents, isolation, lack of social contact, increased dependence, loneliness, and mental health issues were already under pressure (Gardiner et al. 2020; Wang et al. 2024). Apart from the residents themselves, voices from society also challenged the way they were being treated in this pandemic isolation (Backhaus et al. 2021; de Rosa et al. 2022; Noten et al. 2022). All these societal and mental health challenges may negatively impact their dignity, underscoring the need for targeted interventions.

Dignity is one of the core values in the treatment and care of nursing home residents (Sæteren and Nåden 2021). Violating this care as a result of, for example, the increasing complexity of long-term care, ageing populations, and existential care needs can cause distress (Hall et al. 2014). In Canada, Chochinov developed an empirical model that encompasses the various facets of dignity, which resulted in a dignity therapy (Chochinov et al. 2002, 2005). Dignity Therapy is a short, individualized psychotherapy aimed at decreasing the existential distress of patients with a terminal illness. The intervention focuses on enhancing feelings of dignity. “Dignity therapy invites patients to discuss issues that matter most or that they would most wish to be remembered. Sessions are transcribed and edited, with a returned final version that they can bequeath to a friend or family member” (Chochinov et al. 2005). This therapy consists of a guided process by a health professional based on 10 questions, in which patients reflect on important life events, values, and messages they want to leave behind for their loved ones, culminating in a generativity document. Dignity Therapy is based on the Dignity Therapy Question Protocol (DTQP) (Chochinov 2002; Chochinov et al. 2012). Terminal patients with cancer who were offered Dignity Therapy reported an increased sense of dignity and an improved quality of life. Additionally, there was also an effect on reducing depression and increasing self-esteem (Chochinov et al. 2005). The DTQP has been translated and validated for Dutch nursing home residents without cognitive impairments (van Dammen et al. 2025).

Several studies in Canada and England have demonstrated that dignity therapy is beneficial for older people in long-term care as well (Hall et al. 2012). Given that dignity should be an important aspect of care in Dutch nursing homes, also from a human rights perspective (Wijngaart and Witte 2015), dignity therapy can be a valuable addition to supporting dignity. Because of possible cultural and organizational differences as compared to Canada, the aim of this study was to evaluate the feasibility of implementing Dignity Therapy in Dutch nursing homes and to explore its potential effects on residents’ dignity, depression, and self-esteem.

## Methods

### Design

A feasibility study was conducted employing a pre–post design to assess changes over time.

### Setting and participants

The study was conducted in 5 Dutch nursing homes, which are part of 2 larger residential organizations. Through the board of the nursing homes, permission was required to invite residents to participate. Residents with full cognitive capabilities of somatic departments for long-term stays were selected based on the in- and exclusion criteria below, and invited by a psychologist or nursing home physician to participate in the study. Both verbal and written information about the study was provided. If a resident was willing to participate and had signed the informed consent, the resident was included in the study.

Inclusion started in August 2022 and ended in August 2024.

Inclusion criteria for residents were:

- Residing on a ward for long-term residential care.
- Being 18 years of age or older.
- Being cognitively capable of participating, as indicated by the involved psychologist and healthcare professional (nurse or physician).

Exclusion criteria were:

- Not being able to speak or read Dutch.
- Not being able to communicate with the interviewer who administered the questionnaire.
- Being too vulnerable to participate, as indicated by an involved healthcare professional (nurse or physician).
- Not understanding the instructions.
- Not being able to independently decide about participation.

### Outcome measures

To examine the feasibility of the application of Dignity Therapy, this study assessed whether psychologists working in 1 of the 5 participating nursing homes were able to recruit 36 residents willing to participate, the number of residents who needed to be approached to achieve this target, and the reasons for declining participation. Furthermore, the study evaluated whether Dignity Therapy affected residents’ sense of dignity, reduced depressive symptoms, and enhanced self-esteem. Finally, residents’ experiences with the intervention were explored by using a structured 5-point Likert scale questionnaire and 2 open questions.

### Intervention

The interviews were conducted by psychologists. Before the interviews, the researcher (HvD), a psychologist who had completed a 2.5-day training course in dignity therapy with Professor Harvey Max Chochinov in Winnipeg, Canada, covering the theoretical and practical aspects of the therapy, trained all participating psychologists who would perform the interviews.

The interviewer planned an interview of about 45 minutes within 2 weeks after the informed consent was signed. Interviews took place face-to-face in the resident’s room. During this audio-recorded interview, the psychologist used the Dutch Dignity Therapy Question Protocol (DTQP-NL) (van Dammen et al. 2025). After the interview, the interviewer made an extended written summary of the interview, which, as part of the therapy, would be shared with the resident.

### Instruments

At the start of the interview, the background characteristics gender, age, religion and the level of importance of religion on a 5-point scale (very important–not important at all), degree of optimism on a 5-point scale (very optimistic–very pessimistic), highest completed education and number of completed school years, and the number of times per week that family or acquaintances visited them in the nursing home, were requested. Before the interview, directly after having shared the document with the resident, and 8 weeks after having returned the written summary, background information, and data concerning the level of dignity, depressive symptoms, and self-esteem were gathered with the help of questionnaires.

Sixty-one residents were approached to participate in the study. At all 3 time points, the following instruments were administered:

**Dignity impairment**. To determine the level of dignity impairment, the Measurement Instrument for Dignity Amsterdam for Long-Term Care facilities (MIDAM-LTC) (Oosterveld-Vlug et al. 2014) was used. Chochinov also developed and validated a questionnaire to objectively measure the degree of dignity, the Dignity Therapy Inventory (DPI) (Chochinov et al. 2008). The DPI has been translated into Dutch and is called the MIDAM (Vlug et al. 2011). The MIDAM was then translated, validated, and adapted for use in Dutch nursing homes. It is called the MIDAM-LTC (Oosterveld-Vlug et al. 2014) and was used for this research.

The scale contains 31 symptoms or negative experiences, and the resident was asked to indicate on a 5-point scale (0 not to 4, to a high degree) how much these symptoms or negative experiences impacted their sense of dignity. The instrument contains 5 dignity impairment domains: Evaluation of self in relation to others (7 items), Functional status (8 items), Mental state (3 items), Care and situational aspects (5 items), and Long-term care (8 items). The dignity (domain) scores were calculated by summing the items and converted to a 0–100 scale score, with a higher score indicating a higher level of dignity impairment.

**Depressive symptoms** were determined by means of the Geriatric Depression Scale (GDS-15) (Wancata et al. 2006). The scale consists of 15 items, which can be answered with No (0) or Yes (1). After reverse coding 5 items, a total score is calculated, which could range from 0 to 15. A higher score indicates a higher level of depressive symptoms. A total score of 6 or higher indicates a potential depression.

The 10-item Rosenberg Self-Esteem Scale (Rosenberg 1965; Jongenelis et al. 2007) was administered to determine the level of **self-esteem** of the residents. The items were scored on a 0 (strongly agree) to 3 (strongly disagree) scale. A total self-esteem score was calculated after reverse coding 5 out of 10 items. The scale could range from 0 to 30, and a higher score indicates a lower level of self-esteem.

### Participants’ experiences

After the therapy was completed, participants’ experiences were examined using a structured 5-point Likert scale (fully agree–fully disagree), whereby the total of the answers “fully agree” and “agree” was used as a measure of the residents’ experience. The questions asked included: The interview did me good, I enjoyed being interviewed, I would recommend this therapy to others.

Finally, 2 open questions were asked: What did you think of the length of the interview and the document, and what could be done differently?

### Analysis

SPSS version 30.0 was used for the statistical analyses. One-way repeated measures ANOVA was performed to determine whether levels of (subscales of) dignity, depression, and self-esteem changed over the 3 measurement moments. To find whether gender or religion influenced the change in dignity impairment, depression symptoms, and self-esteem over time, 3 × 2 mixed ANOVAs with Bonferroni correction were performed with time (pre, post, follow-up) as the within-subject factor and gender (male/female) or religion (yes/no) as the between-subject factors. For all analyses, a threshold for significance of *α* = 0.05 (95% confidence interval) was used.

Subgroup analyses were exploratory and underpowered.

## Results

### Feasibility

**Figure 1.**
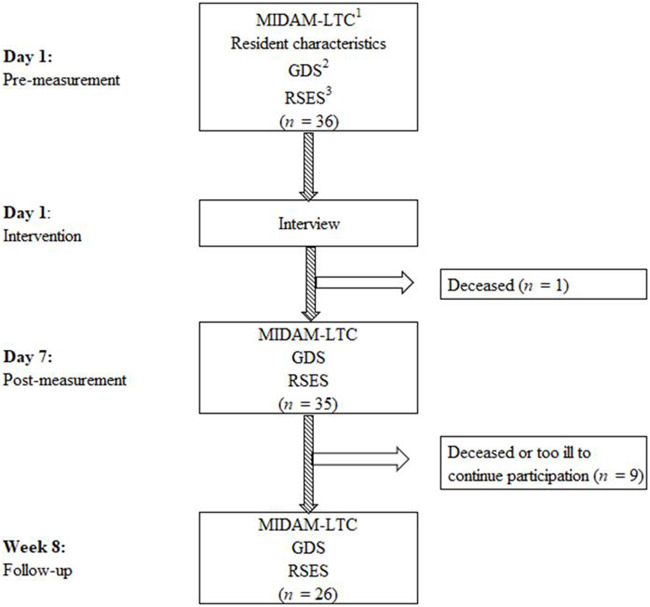
Flowchart of the course of the study.

Thirty-six of the sixty-one invited residents (59%) participated. Due to illness and the natural death of 10 residents, 26 (72%) completed all time points (see Figure 1). Reasons for not participating (*n* = 26) varied from a lack of interest in the study, feeling that their dignity was sufficiently preserved, to suspicion that the results of the study could lead to negative treatment.

At the recruitment of candidates for this study, it was observed that the word “Dignity” raised questions among several residents concerning the meaning of this therapy.

The mean duration of the entire therapy process was 384 minutes (*SD* = 23.73).

### Effectiveness

Table 1 shows participant characteristics. Two-thirds of the participants were female (*n* = 24, 66.7%), and the age of the sample ranged from 60 to 98 (*M* = 83.0, *SD* = 10.1). They had a mean of 2.38 (*SD* = 2.00) visits (from relatives) a week.

**Table 1.** Participant characteristics

**Characteristics**	***N***	**%**
**Gender^a^**		
Male	11	30.6
Female	24	66.7
**Religion^a^**		
Yes	20	55.6
No	15	41.7
**Educational level^a^**		
No education	2	5.6
Primary education	4	11.1
Lower vocational education	6	16.7
Lower secondary education	9	25.0
Secondary vocational education	3	8.3
Higher secondary education	4	11.1
Higher professional education	5	13.9
University	2	5.6
	*M* (*SD*)	Min–max
Age (years)^b^	83 (10.1)	60–98
Optimism^b+c^	2.0 (1.0)	1–5
Years of education^d^	10.8 (3.8)	4–19
Visits family (times per week)^e^	2.4 (2.0)	0–7

a Notes: One participant is missing for gender, religion, or educational level; ^b^Two participants are missing for age and level of optimism; ^c^Optimism is coded in a way that a higher score means a lower level of optimism; ^d^Three participants are missing for years of education; ^e^Four participants are missing for several family visits.

Descriptive statistics of the main variables of the study during the first measurement are shown in Table 2. The highest mean dignity impairment was reported for functional status (*M* = 24.9, *SD* = 21.7). The dignity impairment score was lowest regarding care situational symptoms or negative experiences (*M* = 5.6, *SD* = 10.5). Based on the threshold for depression, 11 participants (30.6%) had a depression score of 6 or higher at the start of the study, indicating a potential depression. More men (*n* = 6, 54.5%) than women (*n* = 5, 20.8%) reported 6 or more depression symptoms; however, this difference was not significant, Fisher’s exact *p* = .062.

**Table 2.** Descriptive statistics of the main variables pre-measurement (*N* = 36)

Variables	*M*	*SD*	Min	Max
Total dignity^a^	16.0	15.5	0.0	71.0
Mental state	14.6	25.1	0.0	100.0
Evaluation	13.8	16.8	0.0	71.4
Functional status	24.9	21.7	0.0	75.0
Care situational	5.6	10.5	0.0	40.0
Long-term care	16.2	19.4	0.0	81.3
Depression^b^	4.1	2.8	0.0	11.0
Self-esteem^c^	20.6	5.5	7.0	29.0

a Notes: MIDAM-LTC; ^b^Geriatric Depression Scale; ^c^Rosenberg Self-Esteem Scale.

Table 3 presents the results, indicating that there were no statistically significant changes over time in mean dignity levels, *F*(1.6, 40.2) = 0.5, *p* = .596, *η_p_*^2^ = .02, depressive symptoms, *F*(2, 46) = 0.8, *p* = .448, *η_p_*^2^ = .03, or self-esteem, *F*(2, 44) = 0.8, *p* = .478, *η_p_*^2^ = .03.

**Table 3.** Change in dignity impairment, depression and self-esteem

	Pre	Post	Follow-up			
Scales	*M* (*SD*)	*M* (*SD*)	*M* (*SD*)	*F*	*p*	*η_p_*^2^
Dignity impairment^a^	53.9 (20.4)	53.0 (21.5)	51.6 (20.2)	0.5	.596	.02
Mental state	4.9 (3.1)	4.3 (2.4)	4.5 (2.6)	1.2	.330	.04
Evaluation	11.7 (5.1)	11.1 (5.3)	11.2 (5.0)	0.5	.610	.02
Functional status	17.2 (7.5)	16.5 (7.3)	15.8 (7.4)	0.7	.479	.03
Care situational	6.2 (2.2)	6.8 (3.8)	6.6 (2.5)	0.5	.550	.02
Long-term care	14.0 (6.8)	14.3 (7.2)	13.7 (6.3)	0.2	.760	.01
Depression^b^	3.7 (2.8)	3.4 (3.0)	3.5 (3.1)	0.8	.448	.03
Self-esteem^c^	20.1 (6.0)	21.0 (5.0)	20.3 (5.4)	0.8	.478	.03

a Notes: As measured by using the MIDAM-LTC; ^b^Geriatric Depression Scale; ^c^Rosenberg Self-Esteem Scale.

**Figure 2.**
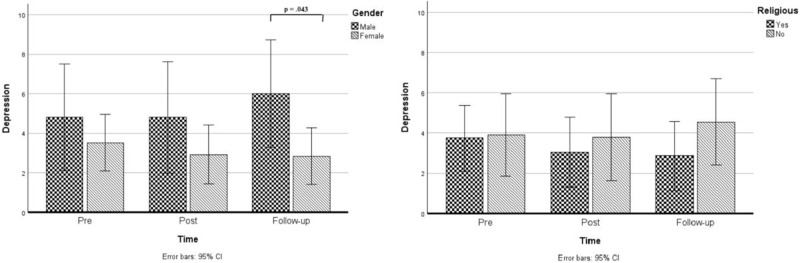
Changes in depression symptoms between pre-, post-, and follow-up measurements for males and females and for religious and non-religious residents.

Using mixed ANOVAs, we tested whether male and female residents showed different trajectories in dignity, depression, and self-esteem over time (Table 4). Concerning dignity impairment, there was no interaction effect between time and gender, *F*(2,46) = 0.2, *p* = .855, *η_p_*^2^ = .01, indicating that the course of dignity impairment over time was comparable between males and females. However, there appeared to be an interaction between time and gender for depression, *F*(2,42) = 4.2, *p* = .021, *η_p_*^2^ = .17. As shown in Figure 2, pairwise comparisons with Bonferroni correction also showed a statistically significant difference in depression symptoms between males (*M* = 6.0 *SD* = 4.7) and females (*M* = 2.8, *SD* = 2.3) during the follow-up measurement whereby the depressive symptoms in men increased, *M*_difference_ = 3.2, *p* = .043, but not during the pre- and post-measurements. No statistically significant differences between male and female residents were found regarding changes in self-esteem over time.

**Table 4.** Means, standard deviations, and mixed ANOVA statistics for dignity impairment, depression and self-esteem for males and females

	Male (*n* = 5)		Female (*n* = 20)		ANOVA				
	*M*	*SD*	*M*	*SD*	Effect	*F*	*df*	*p*	*η_p_*^2^
Dignity impairment									
Pre	24.1	17.7	16.9	16.7	Time	0.0	2, 46	.963	.00
Post	22.9	18.4	17.2	17.4	Gender	0.8	1, 23	.378	.03
Follow-up	24.0	16.6	15.6	16.1	Time × Gender	0.2	2, 46	.855	.01
DI Mental state									
Pre	21.7	27.4	14.6	26.8	Time	1.5	2, 46	.229	.06
Post	11.7	26.1	44.3	19.0	Gender	0.1	1, 23	.797	.00
Follow-up	13.3	19.2	12.5	22.5	Time × Gender	0.4	2, 46	.652	.02
DI Evaluation									
Pre	25.0	23.7	14.7	17.2	Time	0.7	2, 46	.525	.03
Post	26.4	20.8	12.1	18.3	Gender	1.2	1, 23	.276	.05
Follow-up	18.6	14.6	14.7	18.8	Time × Gender	1.6	2, 46	.221	.06
DI Functional status									
Pre	35.0	23.9	24.8	21.3	Time	0.6	2, 46	.560	.03
Post	23.8	18.3	28.8	23.7	Gender	0.1	1, 23	.801	.00
Follow-up	27.5	20.7	24.7	23.9	Time × Gender	1.8	2, 46	.176	.07
DI Care situational									
Pre	10.0	14.6	5.5	10.6	Time	0.9	2, 46	.435	.04
Post	16.0	25.1	7.5	17.9	Gender	1.7	1, 23	.211	.07
Follow-up	17.0	19.9	6.0	10.0	Time × Gender	0.5	2, 46	.638	.02
DI Long-term care									
Pre	22.1	19.2	18.9	22.2	Time	0.4	2, 46	.653	.02
Post	28.1	27.9	18.6	21.6	Gender	1.3	1, 23	.268	.05
Follow-up	33.8	28.3	14.6	15.6	Time × Gender	1.9	2, 46	.161	.08
Depression									
Pre	4.8	4.1	3.5	2.5	Time	1.4	2, 42	.253	.06
Post	4.8	4.4	2.9	2.6	Gender	2.1	1, 21	.158	.09
Follow-up	6.0	4.7	2.8	2.3	Time × Gender	4.2	**2, 42**	**.021**	**.17**
Self-esteem									
Pre	21.2	7.6	19.3	5.5	Time	0.8	2, 40	.446	.04
Post	20.4	7.8	21.0	4.4	Gender	0.0	1, 20	.953	.00
Follow-up	18.6	6.8	20.4	5.0	Time × Gender	1.9	2, 40	.170	.09

Comparing the change in dignity impairment over time between religious and non-religious residents, no interaction effect between time and religion was found, *F*(2, 46) = 0.4, *p* = .707, *η_p_*^2^ = .02 (Table 5). There was a statistically significant interaction effect for depression, *F*(2, 42) = 3.8, *p* = .030, *η_p_*^2^ = .15, indicating that the change in depression levels over time was different between religious and non-religious persons, with religious participants having fewer depressive symptoms than non-religious participants. In the pairwise comparisons with Bonferroni correction, however, no statistically significant differences in depression between religious and non-religious people were found at each of the 3 time points (Figure 2). There was no statistically significant interaction effect regarding self-esteem, *F*(2,40) = 0.4, *p* = .647, *η_p_*^2^ = .02.

**Table 5.** Means, standard deviations, and mixed ANOVA statistics for dignity impairment, depression and self-esteem for religious and non-religious residents

	Religious (*N* = 16)		Not religious (*N* = 9)		ANOVA				
Variable	*M*	*SD*	*M*	*SD*	Effect	*F*	*df*	*p*	*η_p_*^2^
Dignity impairment									
Pre	18.0	18.2	18.9	14.8	Time	0.1	2, 46	.920	.00
Post	17.5	19.1	19.9	14.7	Religious	0.1	1, 23	.709	.01
Follow-up	15.7	16.7	20.0	16.2	Time × Religious	0.4	2, 46	.707	.02
DI Mental state									
Pre	17.2	29.3	13.9	22.1	Time	0.8	2, 46	.464	.03
Post	10.9	20.8	12.0	19.6	Religious	0.0	1, 23	.906	.00
Follow-up	13.0	25.1	12.0	14.5	Time × Religious	0.2	2, 46	.808	.01
DI Evaluation									
Pre	15.2	18.6	19.4	19.4	Time	0.3	2, 46	.740	.01
Post	12.5	20.3	19.3	17.5	Religious	0.3	1, 23	.616	.01
Follow-up	15.4	20.1	15.5	14.1	Time × Religious	0.9	2, 46	.404	.04
DI Functional status									
Pre	23.6	22.1	32.6	.21.0	Time	0.2	2, 46	.839	.01
Post	27.3	25.2	28.5	17.9	Religious	0.5	1, 23	.505	.02
Follow-up	22.7	22.6	29.9	24.2	Time × Religious	0.7	2, 46	.483	.03
DI Care situational									
Pre	6.9	11.5	5.6	11.6	Time	0.6	2, 46	.529	.03
Post	9.4	19.7	8.9	19.7	Religious	0.0	1, 23	.951	.00
Follow-up	7.2	10.8	10.0	16.4	Time × Religious	0.3	2, 46	.704	.01
DI Long-term care									
Pre	22.3	23.5	14.7	16.6	Time	0.2	2, 46	.796	.01
Post	19.7	23.8	21.9	22.0	Religious	0.0	1, 23	.891	.00
Follow-up	15.3	15.9	24.0	25.2	Time × Religious	3.0	2, 46	.062	.11
Depression									
Pre	3.7	2.5	3.9	3.5	Time	1.1	2, 42	.337	.05
Post	3.0	2.8	3.8	3.6	Religious	0.5	1, 21	.502	.02
Follow-up	2.9	2.2	4.6	4.2	Time × Religious	3.8	2, 42	**.030**	.15
Self-esteem									
Pre	19.1	5.7	20.7	6.5	Time	1.1	2, 40	.348	.05
Post	20.3	4.5	21.7	6.1	Religious	0.2	1, 20	.653	.01
Follow-up	19.9	5.1	20.0	6.0	Time × Religious	0.4	2, 40	.647	.02

### Residents’ experiences

A total of 28 residents completed the questionnaires. Most residents (25 of 28; 89%) reported that the interview had been beneficial, and 23 residents (82%) stated that they reported positive experiences with participating in the interview. All but 1 (27 of 28; 96%) indicated that they would recommend Dignity Therapy to others.

Answers to the open-ended question “What did you think of the interview?” reflected a range of experiences, from “Difficult, because you have to reveal yourself” to “Interesting, it woke me up.” Other responses included “Pleasant,” “You have to think carefully before you answer,” and “Good, not too long.” Regarding the length of the interview, participants described it as “Good,” “Good, I was able to say what I wanted,” and “Good, not too long and not too short.”

## Discussion

Thirty-six of the sixty-one invited residents (59%) participated in the Dignity Therapy program, of whom 26 residents (72%) were able to complete the entire program. Reasons for non-completion among the 10 residents included natural death and illness. We found no significant effects of Dignity Therapy on dignity impairment or self-esteem, but a statistically significant interaction was observed for depression between male and female residents at follow-up.

There also appeared to be a statistically significant interaction between religious and non-religious participants, with non-religious residents reporting higher depressive symptoms at the 8-week follow-up. Residents across the sample experienced the therapy as enjoyable and meaningful, suggesting that the intervention may contribute positively to subjective well-being even when objective measures do not detect change.

### Feasibility

Recruitment of 35 residents for participation in the Dignity Therapy study was successfully achieved. However, the study also highlighted an important consideration regarding participant inclusion. At the outset, identifying suitable residents who were both eligible and willing to participate proved challenging. The focus on “dignity” raised questions among several residents concerning the meaning of the term, whether they themselves ever experienced a lack of dignity, and how therapy could contribute to enhancing it. The concept of dignity appeared to be open to multiple interpretations and was a topic of considerable discussion.

The research process was found to be time-intensive for the participating psychologists. Although a conservative pre-study estimate suggested that approximately 5 hours would be required to complete the procedures for 1 participant, this duration was surpassed by most psychologists. This is an important consideration for future implementation and scaling of Dignity Therapy within nursing home settings.

### Effectiveness

The results of this study indicate that Dignity Therapy did not significantly improve in residents’ perceived dignity, depressive symptoms, or self-esteem.

In 2015, the Dutch government launched the Dignity and Pride program to improve the quality of nursing home care, which was expanded in 2018 to allow staff to devote more time and attention to residents. When the COVID-19 pandemic began in 2020, societal concerns arose regarding its negative impact on nursing home residents, which were later confirmed by empirical studies.

Against this background, the ongoing societal and policy focus on dignity and well-being in Dutch nursing homes may have influenced the findings of the present study, conducted between August 2022 and August 2024. Baseline data before the pandemic were unavailable, and the high levels of dignity, self-esteem, and mood observed at study onset may have reflected this increased national attention, potentially resulting in a ceiling effect.

Despite the lack of measurable improvements, residents described their participation in the study as both pleasant and meaningful, likely due to increased interpersonal contact inherent to the study procedures and personal attention they received. Previous comparative studies of Dignity Therapy and counselling interventions have similarly shown that counselling can positively affect residents’ well-being, particularly in reducing depression and anxiety and enhancing resilience.

Although the number of male participants was relatively small (*n* = 11), an increase in depressive symptoms was observed among men at the 8-week follow-up. Several reasons can be cited for this outcome. Firstly, social and cultural norms about masculinity discourage men from expressing their feelings and vulnerability (Addis and Mahalik 2003; Swetlitz 2021). Dignity therapy gives them the opportunity to reflect on this, and could be a reason why unprocessed emotions surface. In addition, women more often use emotion-focused strategies than men (Tamres et al. 2002; Liddon et al. 2018). It is possible that Dignity therapy is more suitable for women and that men feel less helped, which may exacerbate any complaints. To date, there has been little specific research into gender differences in dignity therapy. This raises the question of whether this therapy should be adapted and evaluated for specifically male or female participants.

Various other factors may play a role, including confrontation with loss of social status, identity, etc., due to differences between men and women.

Non-religious participants exhibited higher levels of depressive symptoms at the 8-week follow-up compared to the pre-measurement, whereas this increase was not observed among religious participants. Limited research has examined the influence of religious beliefs on the outcomes of Dignity Therapy. However, existing evidence suggests that spirituality and religion can play a meaningful role in psychotherapeutic processes. A meta-analysis by Bouwhuis-van Keulen et al. demonstrated that person-centered therapy incorporating attention to religious beliefs is particularly effective for patients with depression, whereas non-religious individuals may derive less benefit from such approaches. Similarly, research by Pargament et al. showed that religious coping mechanisms contribute to significant improvements in mental health and emotional adjustment, benefits that may not be accessible to individuals without religious affiliations.

## Strengths and limitations

This study provides the first exploration of the feasibility of implementing Dignity Therapy in Dutch nursing homes. Strengths include the use of validated measurement instruments and the integration of residents’ subjective experiences, which together enhance both internal and ecological validity. The study also offers practical insights into recruitment feasibility, therapist workload, and residents’ reflections on the concept of dignity.

However, several limitations should be acknowledged. The relatively small and selective sample and the absence of a control group limit the generalizability of the findings and preclude firm conclusions about effectiveness. A potential ceiling effect may have occurred, given the high baseline levels of dignity and self-esteem following national initiatives focusing on dignity in care. The 8-week follow-up period may have been too short to detect sustained effects. Furthermore, while exploratory analyses suggested gender- and religion-related differences, these results should be interpreted with caution due to small subgroup sizes. Despite these limitations, the study offers important guidance for the refinement and implementation of Dignity Therapy in long-term care settings.

## Conclusion

This study indicates that Dignity Therapy can be implemented in Dutch nursing homes. Psychologists were able to recruit participants and deliver the intervention as intended. Residents generally evaluated the therapy as pleasant and meaningful. However, no measurable effects were found on residents’ dignity, depressive symptoms, or self-esteem.

The lack of observed changes may be related to high baseline levels of well-being and dignity, possibly reflecting the broader national focus on these values within Dutch nursing home care. The findings suggest that, while Dignity Therapy is acceptable and appreciated by residents, its measurable benefits in this context remain uncertain.

Future research should include residents with lower baseline dignity, larger samples, control conditions, and longer follow-up periods. Studies should also explore whether adaptations may be beneficial for specific subgroups, such as male or non-religious residents.

## Data Availability

The data that support the findings of this study are available on request from the corresponding author. The data are not publicly available due to privacy or ethical restrictions.
