# Feasibility of implementing dignity therapy in Dutch nursing homes: A pre–post study exploring potential effects on dignity, depression, and self-esteem – ERRATUM

**DOI:** 10.1017/S1478951526102132

**Published:** 2026-03-31

**Authors:** Herman Van Dammen, Kris Vissers, Gert-Jan van der Putten, Yvonne Engels

**Keywords:** Dignity therapy, nursing home residents, depression, self-esteem, feasibility, erratum

The Author and Publisher apologise for three author names being accidentally omitted at submission and not corrected subsequently during production. Both the author and the publisher apologise for this error.

The authors and affiliations have since been added to the published article.
